# Acroplate – a modern solution for the treatment of acromioclavicular joint dislocation

**Published:** 2009-04-25

**Authors:** Cîrstoiu C., Rădulescu R., Popescu D., Ene R., Circotă G., Bădiceanu Corina

**Affiliations:** * Orthopedic-Traumatology Clinic of the Universitary Emergency Hospital, Bucharest, Romania

**Keywords:** dislocation, place, pins, osteosynthesis, motion

## Abstract

Two main ways to fix the reduction were imposed in surgical treatment of the acromioclavicular joint dislocations: fixation with trans acromioclavicular pin (Phemister method) and fixation with plate and screws type acroplate. The purpose of the present paper work is to compare immediate and later postoperative results between the two types of surgical interventions. During 2005-2007, 37 surgical reductions and fixation of acromioclavicular joint dislocations were performed in the Orthopedic-Traumatology Clinic of SUUB. In 17 cases a fixation with screws and plates type acroplate has been performed and in 20 cases with pins using the Phemister method. Sex ratio: 31 men and 8 women. Patients were aged between 17 and 56 years old. Follow up at 6 weeks, 3, 6, 12 and 18 post-operatory months. Osteosintesis material removing was done postoperatively, at 4 weeks in case of acroplate's and at 6 weeks in case of the pins. All patients treated of fixation with plate and screws acroplate type had a favorable evolution/development, starting with the shoulder joint mobilization at 24 hours postoperatively, with a complete recovery 4 weeks after the operation, at the same time with the ablation, and without immediate other late complications. As far as the patients treated by using the Phemister method are concerned, they were applied an immobilization, postoperatively. Desault bandage or the scarf for a period between 1 and 3 weeks, beginning with the shoulder joint mobilization later on and a full recovery after a minimum of 6 weeks. However, 3 of the cases showed a migration of one or both pins. Following the study, a more rapid recovery resulted, complete, and without complications of mobility in the shoulder joint, when using plate type acroplate vs pin.

## Introduction

**Fig. 1:**
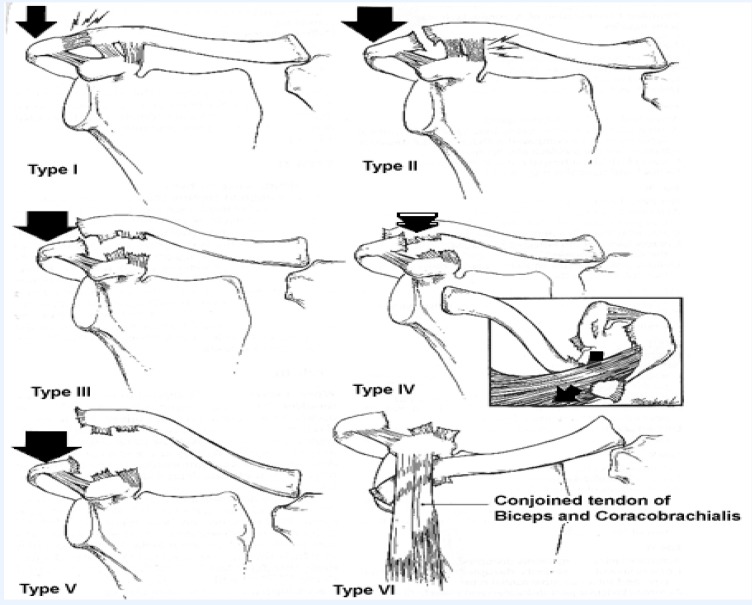
Rockwood classification of the acromioclavicular joint

Acromioclavicular joint dislocations are frequently encountered in young adults, more frequently in men, appearing as a result of sports injuries or accidents at work. The most frequent mechanism is the fall on the shoulder followed by a violent contraction of the sternocleidomastoidian and the Trapeze muscles. The means of content for the joint articulation are the capsule and the acromioclavicular ligament. The other elements which also contribute to the stability of the articulation are the coracoacromial and coracoclavicular ligaments. Depending on their level of injury, the most frequent classification of the acromioclavicular joint dislocations is that of Rockwood (**Fig. 1**, **Table 1**).

**Table 1:** Rockwood classification of the acromioclavicular joint dislocations

1st Degree	Extension of the acromioclavicular ligaments	-clear acromioclavicular articulation	Orthopedic treatment
		-clear coracoclavicular ligaments	
		-clear trapeze and deltoid muscles	
2nd Degree	Break of the acromioclavicular ligaments	-increase the space in acromioclavicular joint	Orthopedic treatment/surgery
		- extended coracoclavicular ligaments	
		-clear trapeze and deltoid muscles	
3rd Degree	Break of the acromioclavicular and coracoclavicular ligaments	- acromioclavicular joint dislocations	Orthopedic treatment
		- increase of the acromioclavicular space with 25-100%	
		- muscles trapeze and deltoid detached of the distal part of clavicle	
4th Degree	Break of the acromioclavicular and coracoclavicular ligaments	- coracoclavicular space increased/normal	Surgery treatment
	Acromioclavicular joint dislocations with posterior movement of the clavicle	- trapeze and deltoid muscles detached	
5th Degree	Break of the acromioclavicular and coracoclavicular ligaments	- increase of the acromioclavicular space with 100-300%	Surgery treatment
	Acromioclavicular joint dislocations	- trapeze and deltoid muscles detached	
6th Degree	Break of the acromioclavicular ligaments	Subcoracoidal type	Surgery treatment
		- distal clavicle is dislocated inferior to coracoids process	
		- coracobrachialis ligaments are disrupted	
		Subacromial type	
		- clavicle inferior to acromion	
		- coracoclavicular space is smaller	
		- coracoclavicular ligaments are disrupted	

Depending on its degree, the treatment of the acromioclavicular joint dislocations could be orthopedic (immobilized on scarf-1st degree and Robert-Jones bandage –2nd degree) or surgically. Several surgical techniques were described as treatment for recent acromioclavicular joint dislocations; two main methods were imposed in our clinic for their reduction fixation: fixation with transacromioclavicular pins (Phemister method) and fixation with plate and screw type acroplate. The aim of the present paper work is to compare immediate and later postoperative results between the two types of surgery interventions.

## Materials and method

**Fig. 2:**
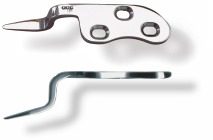
Plate type acropolate which was used

During 2005-2007, 37 acromioclavicular joint dislocations were performed in the Orthopedic-Traumatology Clinic of SUUB. Of these, 11 were acromioclavicular joint dislocations of 2nd degree, 19 were acromioclavicular joint dislocations of 3rd degree, 5 were acromioclavicular joint dislocations of 4th degree, and 2 were acromioclavicular joint dislocations of 5th degree. In 17 cases a fixation with screws and plate type acroplate has been used and in 20 cases one with pins using Phemister method. The surgeon chose the method of fixation depending on disjunction degree. Sex ratio was 31 men and 8 women. Patients were aged between 17 and 56 years old. They used a plate with 3 screws acroplate type and long hook, with anatomic profile for the right and the left shoulder (**Fig. 2**). The tooth plate which entered in the back of acromion, was performed a luxation and was fixed with 3 screw plates at the top of the clavicle. Moreover, the acromioclavicular and coracoclavicular ligaments were also welded.

The benefits of the plates consisted of positioning the plate under Fascia muscle that covers the deltoid and the trapeze muscles and then positioning the hook in the back part of the shoulder joint articulation, in order to prevent a possible subacromial conflict. This allowed material ablation without damaging the acromioclavicular ligaments, the hook’s angle being adjusted at 15-20 degrees to the acromioclavicular angle. A short incision that avoided the violation of the supraclavicular nerve allowed a satisfactory cosmetic result.

**Fig. 3:**
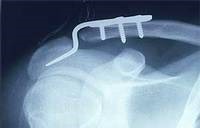
Post-surgery radiography

All interventions went on without intraoperative or immediate postoperative actions. Patients have carried out regular clinical and radiological examinations at 6 weeks, 3, 6, 12 and 18 months postoperatively. Ablation of the osteosynthesis material was done 4 weeks postoperatively in the case of acroplates and 6 weeks in the case of pins.

## Results

All the patients treated of fixation with plate and screws acroplate type had immediate, but also later favorable evolution. Shoulder joint mobilization was done 24 hours postoperatively, then balance movements of the arm, and finally moves of flexural-extension and active and passive rotation of the shoulder joint. During the 4 weeks, a limitation of the abduction movement of the arm up to 90 degrees was observed. This limitation was explained by the presence of the tooth plate in the back of the acromion. After 4 weeks, a complete recovery was observed together with the ablation plate. There were no immediate or later complications.

Regarding the patients treated with the Phemister method, applied to post-immobilization Desault bandage or the scarf for a period of between 1 and 3 weeks. Thus, starting of shoulder joint mobilization was late with a full recovery after minimum 6 weeks. In 3 cases there were later complications and the migration has been observed at one or both pins. 

## Conclusions

The Acroplate provides a stable and firm fixation of acromioclavicular joint dislocation, without hindering the healing of the ligaments. Provides a primary stability and allows early mobilization, as well as the starting of early recovery exercises of shoulder muscles.

Following the study we noticed a more rapid recovery, without complications and complete mobility in the shoulder joint when using plate type acroplate vspin. Thus we consider it now the most effective solution in the surgical treatment of acromioclavicular joint dislocation.
